# Fast and optimal algorithm for case-control matching using registry data: application on the antibiotics use of colorectal cancer patients

**DOI:** 10.1186/s12874-021-01256-3

**Published:** 2021-04-02

**Authors:** Pavlos Mamouris, Vahid Nassiri, Geert Molenberghs, Marjan van den Akker, Joep van der Meer, Bert Vaes

**Affiliations:** 1 Department of Public Health and Primary Care, KU Leuven, Kapucijnenvoer 33, J building, 3000 Leuven, Belgium; 2 Open Analytics NV, Antwerp, Belgium; 3 I-BioStat, KU Leuven University of Leuven, Leuven, Belgium; 4 I-BioStat, Hasselt University, Diepenbeek, Belgium; 5 Department of Family Medicine, Care and Public Health Research Institute, Maastricht University, Maastricht, the Netherlands; 6 Institute of General Practice, Goethe University, Frankfurt am Main, Frankfurt, Germany

**Keywords:** Case-control, Optimal matching, Comorbidity index, Colorectal cancer

## Abstract

**Background:**

In case-control studies most algorithms allow the controls to be sampled several times, which is not always optimal. If many controls are available and adjustment for several covariates is necessary, matching without replacement might increase statistical efficiency. Comparing similar units when having observational data is of utter importance, since confounding and selection bias is present. The aim was twofold, firstly to create a method that accommodates the option that a control is not resampled, and second, to display several scenarios that identify changes of Odds Ratios (ORs) while increasing the balance of the matched sample.

**Methods:**

The algorithm was derived in an iterative way starting from the pre-processing steps to derive the data until its application in a study to investigate the risk of antibiotics on colorectal cancer in the INTEGO registry (Flanders, Belgium). Different scenarios were developed to investigate the fluctuation of ORs using the combination of exact and varying variables with or without replacement of controls. To achieve balance in the population, we introduced the Comorbidity Index (CI) variable, which is the sum of chronic diseases as a means to have comparable units for drawing valid associations.

**Results:**

This algorithm is fast and optimal. We simulated data and demonstrated that the run-time of matching even with millions of patients is minimal. Optimal, since the closest controls is always captured (using the appropriate ordering and by creating some auxiliary variables), and in the scenario that a case has only one control, we assure that this control will be matched to this case, thus maximizing the cases to be used in the analysis. In total, 72 different scenarios were displayed indicating the fluctuation of ORs, and revealing patterns, especially a drop when balancing the population.

**Conclusions:**

We created an optimal and computationally efficient algorithm to derive a matched case-control sample with and without replacement of controls. The code and the functions are publicly available as an open source in an R package. Finally, we emphasize the importance of displaying several scenarios and assess the difference of ORs while using an index to balance population in observational data.

**Supplementary Information:**

The online version contains supplementary material available at 10.1186/s12874-021-01256-3.

## Background

Randomized controlled trials (RCTs) remain the gold standard for assessing intervention efficacy; nevertheless, they are often unable to be generalized due to not including “real world data” on combinations of heterogeneous patients or interventions [1]. In addition, cost, small sample size and long-time duration make trials infeasible for a range of clinical questions.

Observational studies might complement RCTs in hypothesis generation or even establishing questions for future RCTs [2, 3]. However, due to non-randomization, they are more prone to bias, which can be addressed through careful study design and appropriate statistical analysis [4]. Observational studies include, among others, the cohort and the case-control studies [3]. This paper focuses on the latter.

Studies with a case-control design can be conducted and completed in a shorter time compared to those with a cohort design [5]. Such studies require smaller sample sizes and hence are usually less expensive. In general, it is the only practical approach for identifying risk factors for rare diseases, especially where follow-up of a large sample for occurrence of the condition might be impractical [6]. In a case-control setting, subjects with a disease or a condition (cases), are matched to subjects without the disease (controls) in order to create similar groups in terms of confounding variables [7]. The purpose of case-control studies is to retrospectively identify risk-factors and investigate the association between exposure and outcome [8]. An important aspect of case-control studies is the method of sampling controls, more specifically whether a control is sampled with or without replacement, the matching is done greedy or optimally, and whether different sources of bias are avoided [9].

First, in case-control studies a common issue is confounding, where researchers deploy matching in an attempt to ensure comparability between cases and controls and reduce systematic differences due to background variables [10, 11]. Mostly, cases and controls are matched with baseline variables including age and sex and then a matched statistical analysis is performed like conditional logistic regression to draw associations [10]. In registries, where the data is even more diverse, the necessity of balancing the population is of utter importance [12].

Second, a question rises on whether one should sample controls with or without replacement. Sampling with replacement allows the control to be sampled several times, whereas sampling without replacement requires the control to be sampled only once. While incidence density sampling is mostly deployed with replacement of control [13], selection without replacement only produces slight bias [14], especially when there is a great availability of controls to pool. In settings where there are many controls available and there is a need for adjustment on several potential confounding factors, it would be a sensible strategy to limit the opportunity of resampling the same individual, thus increasing the statistical efficiency when adjusting for these predictors [15].

Computationally, sampling with replacement is straightforward and has been implemented before in statistical software including SAS [13], R [16] and via the sttocc command in STATA. The controls are selected randomly, so not always the closest (optimal) control is matched to a case. Sampling without replacement has not been investigated in depth. Nevertheless, there is an approach presented in a SAS conference [17], however it fails to get the closest control since the ordering is occurring on the level of the control.

Therefore, the objective of this paper was twofold, first to generate an optimal, efficient and fast algorithm, which can create a matched-case sample (with and without replacement), assuring that the closest available control is selected (optimal). The algorithm was deployed using the R software [18]. We applied the proposed algorithm in a clinical case using registry data. We investigated the association between the prescription of oral antibiotics and colorectal cancer. In addition, to increase balance in the population, we introduced the Comorbidity Index (CI) variable, which is the sum of chronic diseases as a means to have comparable units for drawing valid associations.

## Methods

### Intego database

Intego is a general practice-based morbidity registration network coordinated at the Department of General Practice of the University of Leuven, Belgium [19]. General practitioners record continuously patient information about baseline characteristics, medications, diagnoses, vaccinations and laboratory tests. The data is extracted using the medical software programme Medidoc (Corilus NV, Aalter, Belgium) [20]. Up to date, there are over 6 million diagnoses, 60 million laboratory results and 22 million medication prescriptions.

Intego procedures were approved by the ethical review board of the Medical School of the University of Leuven (ML 1723) and by the Belgian Privacy Commission (SCSZG/13/079). Only the data of the practices with an optimal registration performance were included in the database [21]. The coding system is universal; medications were classified according to the WHO’s Anatomical Therapeutic Chemical classification system, whereas diagnoses were linked to the International Classification of Primary Care (ICPC-2) and International Statistical Classification of Diseases and Related Health Problems 10th Revision (ICD-10).

### Clinical case definition

#### Study design and study population

We conducted a population-based, case-control study nested within Intego, using data collected from 2000 to 2015. All patients aged above 18 years old between 2010 and 2015 with at least 1 year of follow-up in Intego were eligible. The clinical research question is to investigate the association between colorectal cancer (CRC) and prescription of oral antibiotics.

#### Case selection

Cases were defined as the patients with a registration of a new diagnosis (incidence) of CRC in the period 01/01/2010 till 31/12/2015.

#### Control selection

For each case, the pool of controls consisted of all eligible individuals without a diagnosis of CRC when the case had his or her CRC. Controls were assigned the same index year as their matched case.

#### Exposure

The exposure was the prescription of oral antibiotics up to 10 years prior to the index year.

#### Matching variables

Table 1 provides an overview of the matching variables used in the analysis. The exact variables that we matched were gender, practice and yearly contact group (JCG). The JCG is defined as the year in which patients consult their general practitioner (GP). Age was a varying variable, whereas follow-up was either exact or trimmed. Exact follow-up occurs when cases and controls have exactly the same years of follow-up, whereas trimmed follow-up occurs when the controls have more or equal years of follow-up than the cases. Consider a case that has CRC in year 2010, and the entry date in the registry is 2004. Controls with exact follow-up should enter in year 2004, whereas those with trimmed follow-up could enter before (and including) 2004.

**Table 1:** Variables used in the analysis

Variables	Type	Meaning
Gender	Exact	Gender of patient (Males, Females)
Practice	Exact	Practice ID
JCG	Exact	Year of visiting the GP
Age	Varying	Age of the patient
Follow-up	Exact	Cases and controls should enter in database the same year
Follow-up	Trimmed	Controls could enter in database prior to the case
Comorbidity Index	Exact	Cases and controls should have the same number of diseases
Comorbidity Index	Varying	Controls could have less, equal or more diseases based on a threashold

Comorbidity Index (CI) was considered either exact or varying. Exact CI is defined as the equal number of chronic diseases between cases and controls, whereas continuous is defined with a threshold. Suppose in year 2010, we have a case with 3 chronic diseases, thus CI = 3. When matching occurs using categorical CI, we force the controls to have also CI = 3, however when continuous CI was used, then we allowed for an absolute difference of one disease, meaning that the controls could have 2, 3 or 4 diseases.

#### Statistical analysis

Conditional logistic regression was used to estimate the adjusted odds ratios (ORs) and associated 95% confidence intervals (CIs) to investigate the association between CRC and prescription of oral antibiotics.

### Optimal case-control matching

#### Greedy and optimal matching algorithm

Matching is a standard method to adjust for confounding in observational studies. Rosenbaum [22] has introduced a distance measure *D*_*ij*_ between the *i*^*th*^ case and the *j*^*th*^ potential control.

Let X^1^ = { ${x}_1^1,{x}_2^1,\dots, {x}_p^1$ } and X^0^ = { ${x}_1^0,{x}_2^0,\dots, {x}_p^0$ } be the vector of matching variables for N cases and M controls (M ≥ N). Then, one definition for *D*_*ij*_ is based on the weighted sum of the absolute differences between the *i*^*th*^ case and the *j*^*th*^ potential control, i.e.,

$$ {D}_{ij}=\sum \limits_{k=1}^p\left|{x}_{ik}^1-{x}_{ik}^0\ \right|\times {W}_k $$

The total distance $T=\sum \limits_{i=1}^N{D}_{ij}$ is a natural way to evaluate how well the entire group of cases is matched to the controls.

Two algorithms to compute this distance measure exist, the optimal and the greedy algorithm [22]. The greedy algorithm sorts randomly the cases and controls and matches the first case with the closest control using the smallest distance *D*_*ij*_. This process is repeated until all cases are matched. This algorithm produces good matches but does not guarantee to minimize the total distance T. The optimal algorithm on the other hand, produces the optimal set of matches based on minimizing T. Refer to Rosenbaum [22] for detailed discussions on these algorithms and their properties. In this work, the ccoptimalmatch package performs both, greedy and optimal algorithm in a fast, efficient and reproducible way.

#### Pre-processing steps

The following steps are necessary to derive the dataset in a format that can be used by the algorithm.

(i) Match on exact variables:

The first step is matching upon exact variables, which splits the original dataset into smaller parts. By shifting the analysis from one big dataset to several small subsets, the computational burden decreased substantially. A subset was defined as the factorial combination of the exact variables, namely all the combination of gender, practice and JCG that had at least one case. For example, subset one contains females that visited practice A in year 2010, subset two contains females that visited practice A in year 2011 up to subset 383, which is the last factorial combination of the exact variables. The current clinical case showed 1718 cases and 224,909 eligible controls. After exact matching on gender, JCG and practice, the number of eligible controls dropped to 175,018.

(ii) Create artificial observations and select the range of variables:

Artificial observations for controls were created in all 383 subsets, thus all cases could be matched with all possible controls. More specifically, subset one had two cases and 2217 controls. After creating artificial observations for controls, there were two cases and 4434 controls, thus each case had 2217 controls available to pool. Since our analysis was done on the subset level, it is imperative to have all available controls that are eligible for all cases. The range that was used for specific variables was based upon clinical advice and upon the specific research question. For some research questions age should be as close as possible (e.g., 1-year absolute difference), while for others the age could vary more (e.g., 5 years absolute difference).

(iii) Create the variables “total controls per case” and “frequency of controls”:

The variable “total controls per case” depicts the total pool of controls available for each case, whereas the variable “frequency of controls” depicts how many times a control was assigned to a case. Both variables were essential for constructing the algorithm. The variable “total controls per case” was necessary in order to assign the control to the case that had the least number of controls to pool from. The “frequency of controls” variable was required, since the controls with the lowest frequency were matched first, leaving the controls with the highest frequency available for the next cases.

(iv) Order variables:

Ordering the variables in a correct order was of utter importance. Suppose that there are three variables, namely “age difference”, “follow-up difference” and “frequency of controls”. The dataset should be ordered by the variables “case”, “control”, “follow-up difference”, “age difference” and lastly by “frequency of controls”. The variable “follow-up difference” is ordered before “age difference” since the “follow-up difference” has more weight (importance) than the “age difference”. However, the researcher can switch the order if age has more importance. Ordering by the “frequency of controls” ensures that the controls with the lowest frequency are matched first. This last step is very important since the closest control (optimal) will be available first for each case.

#### Algorithm steps

**Fig. 1:**
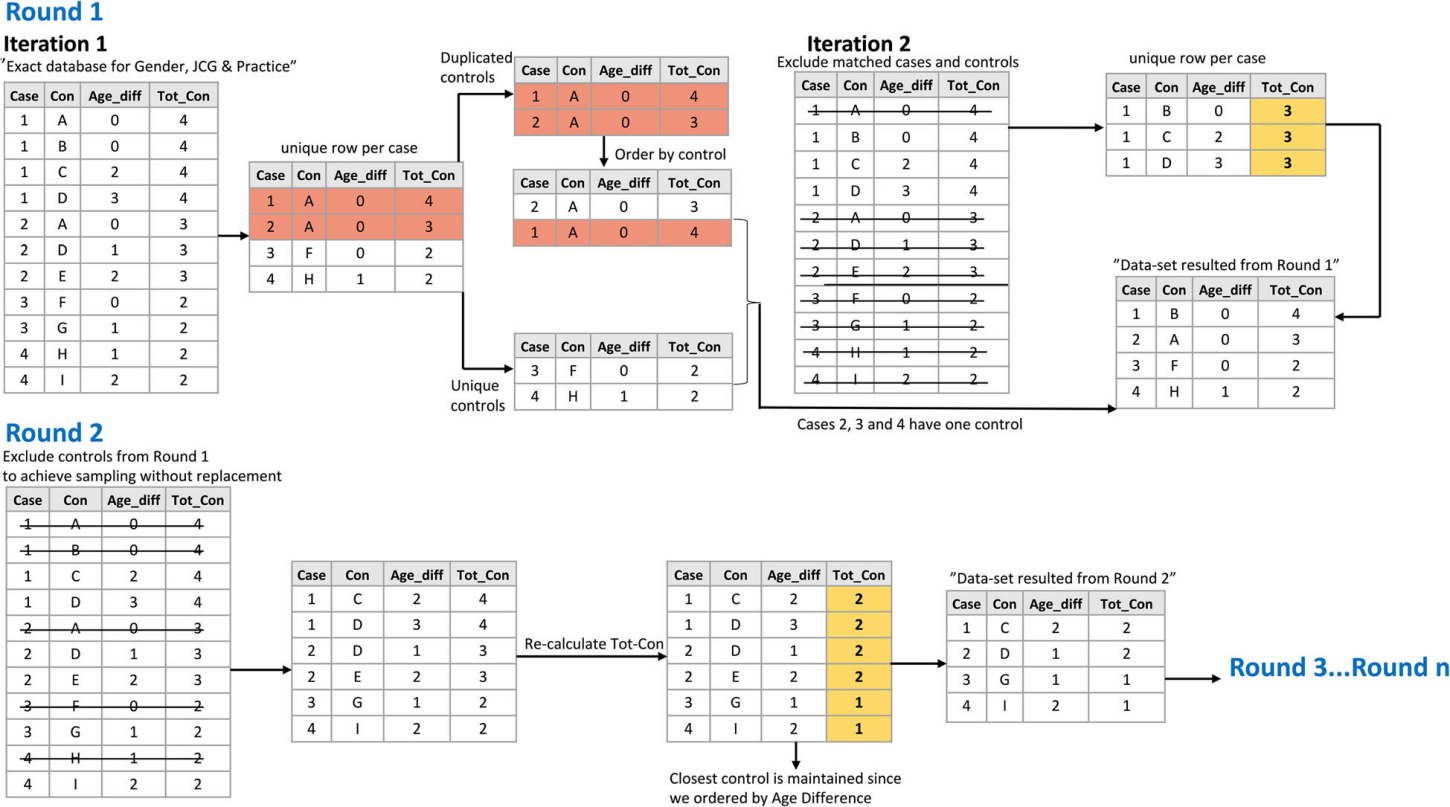
Panorama of optimal case-control matching algorithm

For demonstration purposes, we consider a small dataset with 4 cases and 9 controls as depicted in Fig. 1. This dataset was derived based on the pre-processing steps that we described above. We used exact matching on 3 variables, namely gender, JCG and Practice Id, and the only varying variable is Age_diff, which depicts the age difference between cases and controls.

(i) Start of round 1: Select one control per iteration:

Since the data was ordered, selection of one control per iteration was performed. Therefore, we ascertained that every case had at least one control, and the closest (optimal) control was obtained.

(ii) Split between duplicated and unique controls:

Duplicated controls are assigned to more than one case, whereas unique controls are assigned only to one case. When matching controls with replacement, the algorithm was straightforward since the same control could be matched to several cases. When matching controls without replacement, duplicated controls needed to be assigned optimally to each case. The dataset was split to cases with unique controls and duplicated controls. In the dataset with the duplicated controls, ordering the variable “total controls per case” and assigning the control to that case which had the least pool of controls available assured the optimal matching.

(iii) Exclude matched controls and cases:

When matching without replacement, cases and controls that were already matched were removed.

(iv) Repeat steps (i)-(iii) m times:

The variable “total controls per case” was recalculated and the same iteration-framework as in steps (i)-(iii) was repeated to find the controls for the remaining cases. These iterations proceeded until each case had at least one control (when available).

(v) End of round 1. Continue to round 2 up to round n:

n stands for the number of controls that the user wants to match. If four controls are needed, then there will be four rounds, if ten controls are needed then there will be ten rounds.

## Results

### Algorithm implementation in ccoptimalmatch R package

The algorithm described before, is implemented using the functions of the ccoptimalmatch R package (Additional file 1). A detailed vignette (Additional file 2) is available in the supplementary materials to demonstrate the transformation of the raw, pre-processed data (Additional file 3) to the processed data (Additional file 4), where the optimal algorithm is applied. A real-life dataset of the Intego registry is used, with cases being the patients with CRC and controls without CRC.

Table 2 presents the computational efficiency of the algorithm. Two different scenarios are detailed. We selected a 1:4 case-control ratio, since this is the most used ratio in clinical practice. First, we increased both the cases and controls and inspected the runtime. The first simulation was with 10 cases and 90 controls and the run-time was only 0,33 s, whereas the last simulation was with 100,000 cases and 900,000 controls and the run-time was 1 min. Second, we only increased the controls, whereas the cases remained the same (1000). Again, even with 10,000,000 patients, the algorithm only took 2 min. For each simulation, 100 iterations were used, and the average run-time is displayed.

**Table 2:** Algorithm’s run-time using different simulations

Case-Controls	Total patients	Cases	Controls	Iterations	Run-time
1:4	100	10	90	100	0,33 s
1:4	1000	100	900	100	0,35 s
1:4	10,000	1000	9000	100	0,87 s
1:4	100,000	10,000	90,000	100	6,25 s
1:4	1,000,000	100,000	900,000	100	1 min, 3 s
1:4	10,000	1000	9000	100	0,89 s
1:4	50,000	1000	49,000	100	1,1 s
1:4	100,000	1000	99,000	100	1,6 s
1:4	500,000	1000	499,000	100	6,3 s
1:4	1,000,000	1000	999,000	100	12,7 s
1:4	10,000,000	1000	9,999,000	100	2 min, 7 s

### Fluctuation of ORs

**Fig. 2:**
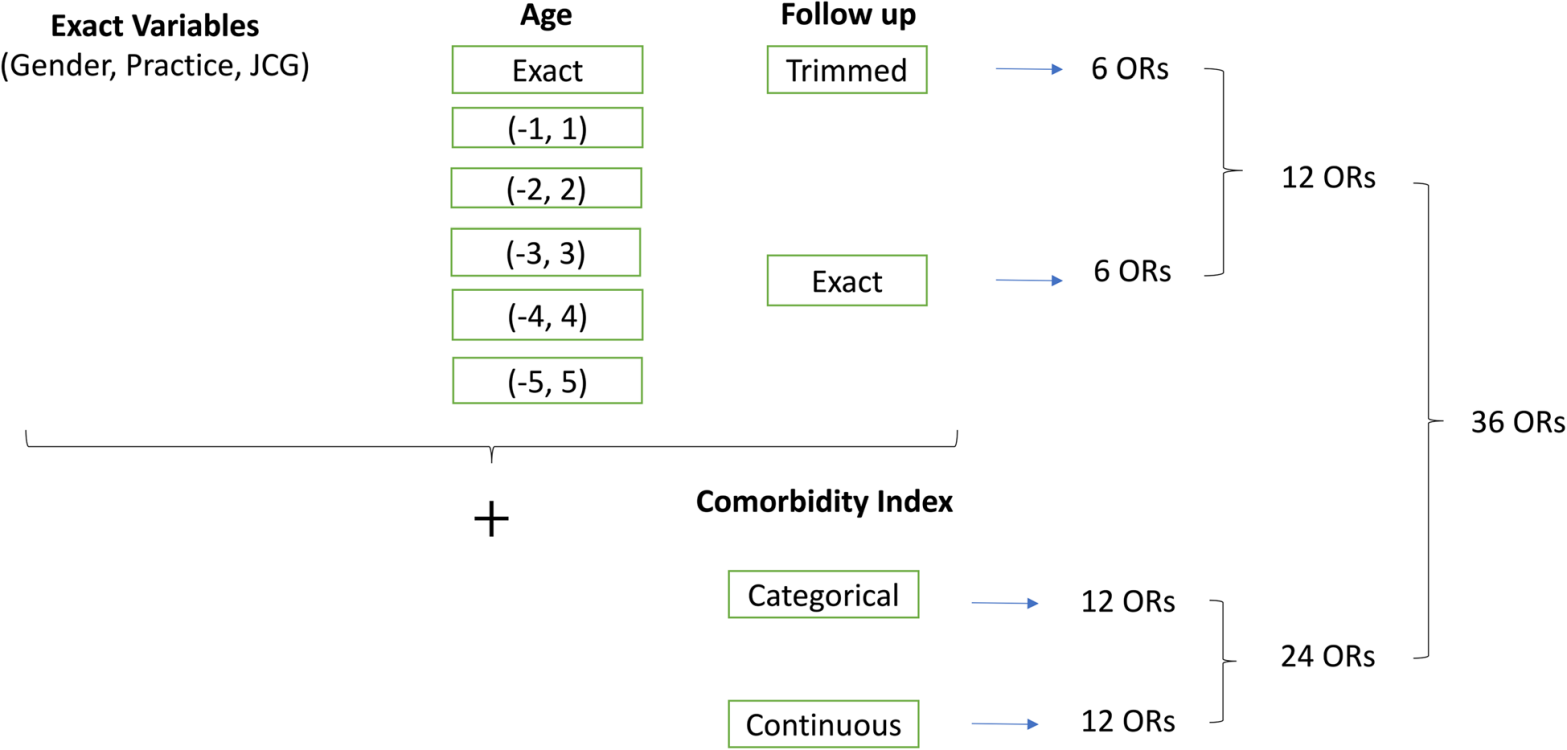
72 combinations of different scenarios using with and without replacement of controls

First, analyses were conducted with replacement of controls, and second without replacement of controls. Four eligible control subjects were optimally matched with each case. In total, 72 scenarios were considered (36 with and 36 without replacement of controls) using the factorial combinations of exact variables, age (6 categories), follow-up (2 categories) and CI (2 categories), as displayed in Fig. 2.

**Fig. 3:**
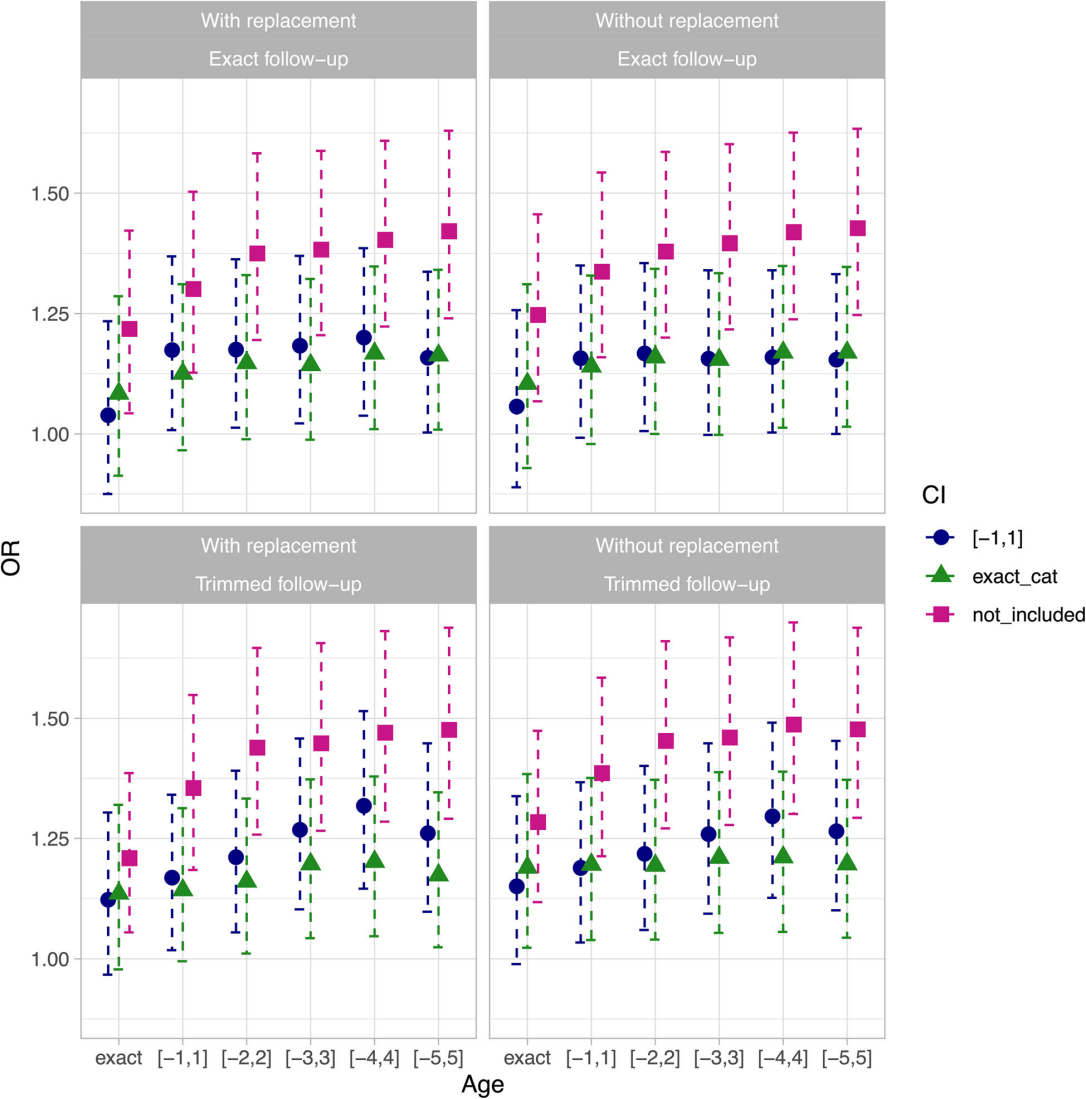
72 odds ratios for the different scenarios

Figure 3 displays the ORs with and without replacement of controls. For both sampling techniques the OR’s were very close. However, when the CI was taken into account, and the population was more balanced, the OR’s decreased substantially. Furthermore, as the age range increased, the OR’s were higher when the CI was not used, yet they fluctuated when the CI was used. When exact follow-up was used, the ORs were slightly lower compared to when the trimmed follow-up was used.

Additional file 5 captures the same information as Fig. 3, yet more detailed. It demonstrates how many controls were sampled for each scenario, and how many cases had one, two, three and four controls respectively. In addition, the ORs are displayed with their 95% confidence intervals. When we relaxed the age range, we observed that the cases matched with only one control substantially reduced since we had more available controls to pool. With the replacement of controls approach, we had more controls available, although not considerably higher, since the number of available controls to pool was very high.

## Discussion

This algorithm is innovative since the controls were not assigned to the cases randomly, but optimally. First, we matched on exact variables, thus creating subsets, which reduced the computational burden. In addition, artificial observations for controls were used; allowing all cases to be combined with all possible controls for optimal matching. Furthermore, we defined the acceptable range of specific variables and ordered them according to an appropriate ordering. Like this, we assured that the closest control would be matched to the case. As a result, if a case had only one control available, we matched this control to that case, thus maximizing the number of cases that could be included in the analysis. Finally, the algorithm generated a matched case-control sample with and without replacement, thus allowing the researcher to choose the method that better fits his interests.

We also investigated the computational efficiency. In our clinical application with 1637 cases and 75,473 controls, the run-time was 0.5 s regardless of how many variables we wished to match and regardless of the scenario we used. We also demonstrated using different simulations, that the run-time is minimal even if there are millions of patients in the database, where the run-time was approximately 2 min.

The ccoptimalmatch R package was created accompanied with a detailed vignette using a real-life dataset (which is also made publicly available for interested researchers), which explains the different steps from the pre-processing phase until the actual application. Therefore, the researcher has a tool to quickly replicate and adjust the code to his needs.

To demonstrate the algorithm, a case study was analysed using a large morbidity registry database in Belgium. From a clinical perspective, including gender, practice, JCG and age as matching variables seems a natural choice, but in retrospective studies the follow-up variable is also crucial. When controls have been longer registered in the database for example, they might have been longer exposed than cases. Therefore, the investigation of the results based on the follow-up time seems an interesting choice.

We also included the comorbidity index (CI) as a matching variable to achieve balance in the disease status of cases and controls (balance population). Since our data is observational, and we wanted to make valid comparisons, our population had to be as close as possible in “risk terms”. Especially in registry data, where the population is very diverse, the necessity of balancing the cases and the controls is highly recommended. Like this, we assured that a severely ill patient was not matched to a healthy control, and vice versa. We observed that when the CI was used, the ORs were considerably lower and very similar (almost identical) to previous research [23, 24]. Our proposed optimal matching algorithm would allow including as many covariates as required to create an acceptable level of balancedness. Advantages of using electronic health record (EHR) data include the longitudinal data, a big sample size and the richness of the data source.

There are some limitations of our work. First, the step of creating artificial observations could require a large memory capacity when the numbers of cases and controls is very high. A feasible solution is that the loop of creating artificial observations is not applied in the whole data set, but rather in subsets. Second, we analysed a clinical case using registry data. Our method, similar to any other method applied on such data, inherits the drawbacks of registry data including the missing data from hospitals, specialists and from basic parameters (e.g., smoking variable). In addition, there could be a delay between the actual outcome and the time of registration. Finally, the entry date and exit date of the patient cannot be identified exactly, thus we had to match on the year, and consequently have less accuracy in the follow-up.

## Conclusion

In this work, we developed an algorithm that takes into account sampling of controls with or without replacement, in a fast, efficient, reproducible and optimal way. Fast, as the simulations demonstrated, since the run-time of matching was minimal even with millions of patients. Reproducible, since an R package has been developed accompanied with a detailed vignette and real-life data from a Belgian registry. Efficient, since it accommodates replacement with or without controls, using as many covariates as the researcher deems necessary and easily applicable. Optimal, since the closest control is always captured and in the scenario that a case has only one control, we assure that this control will be matched to this case, thus maximizing the cases to be used in the analysis.

Building on this algorithm, and since our data is observational, we have illustrated the importance of creating several scenarios and assessing the difference of ORs when using an index to balance population in observational data. The display of these scenarios could provide more robust results, reveal patterns of associations and facilitate communication between researchers.

## Supplementary Information

**Additional file 1.** The functions of the ccoptimalmatch package.

**Additional file 2.** A detailed vignette of the ccoptimalmatch package using a real-life dataset of INTEGO registry (Flanders, Belgium). The pre-processing phase of the algorithm and its application in the clinical case is provided.

**Additional file 3.** The pre-processed data that are used in our study.

**Additional file 4.** The processed data that are used in our study. Those are the data that have been created after applying the pre-processing steps.

**Additional file 5.** ORs and CIs for a factorial combination of variables using different control sampling. 72 Scenarios displaying the ORs and the corresponding CIs for a factorial combination of variables using with and without replacement of controls.

## Data Availability

The datasets, codes and vignette of this manuscript are available as additional files.

## Abbreviations

CI: Comorbidity Index
RCTs: Randomized controlled trials
CRC: Colorectal cancer
JCG: Year of contact
GP: General Pratitioner
ORs: Odds ratios
CIs: Confidence intervals
EHR: Electronic health record
