# Supplementary material for: Fast and optimal algorithm for case-control matching using registry data: application on the antibiotics use of colorectal cancer patients
Source: BMC Med Res Methodol. 2021 Apr 2;21:62. doi: 10.1186/s12874-021-01256-3 (PMC8019172; doi:10.1186/s12874-021-01256-3)
Supplement: Supplementary file 1 — Additional file 1. The functions of the ccoptimalmatch package. [file 12874_2021_1256_MOESM1_ESM.docx]

**Fast and optimal algorithm for case-control matching using registry data: Application on the antibiotics use of colorectal cancer patients**

Pavlos Mamouris^1*^; Vahid Nassiri^2^ ; Geert Molenberghs^3,4;^ Marjan van den Akker^1,5,6;^ Joep van den Meer^1^; Bert Vaes^1^

1. **Function to calculate the iterations per round**

iterations_per_round <- function (dataset, cluster_var, Id_Patient, total_cont_per_case, case_control, mat_per_case = NULL) {

cluster_var <- enquo(cluster_var)

Id_Patient <- enquo(Id_Patient)

total_cont_per_case <- enquo(total_cont_per_case)

one_to_one = dataset %>% group_by((!!cluster_var)) %>% filter(row_number() <= 2)

dup_con = one_to_one %>% distinct() %>% group_by((!!Id_Patient)) %>% filter(n() > 1) %>% arrange((!!Id_Patient), (!!total_cont_per_case)) %>% filter(row_number() ==

1)

if (nrow(dup_con) > 0) {

one_to_one <- anti_join(one_to_one, dup_con, by = quo_name(Id_Patient)) # library(rlang) is needed

one_to_one <- bind_rows(one_to_one, dup_con) %>% arrange((!!cluster_var), case_control)

one_to_one <- one_to_one %>% group_by((!!cluster_var)) %>% mutate(mat_per_case = n() - 1)

case_cntrl_1st_wave <- one_to_one %>% filter(mat_per_case == 1) %>% select(-mat_per_case)

dataset <- anti_join(dataset, case_cntrl_1st_wave, by = quo_name(cluster_var))

dataset <- anti_join(dataset, case_cntrl_1st_wave, by = quo_name(Id_Patient))

} else {

one_to_one <- one_to_one %>% group_by((!!cluster_var)) %>% mutate(mat_per_case = n() - 1)

case_cntrl_1st_wave <- one_to_one %>% filter(mat_per_case == 1) %>% select(-mat_per_case)

dataset <- NULL

}

return(list(case_cntrl_1st_wave = case_cntrl_1st_wave, dataset = dataset, dup_con = dup_con))

}

1. **Function to calculate the optimal matching**

#' optimal_matching

#'

#' optimal_matching is performing the optimal match between cases and controls in an iterative way and

#' computational efficient way

#'

#' Here is where I should put all my details. This is where I should give more examples if necessary

#'

#' @param total_database a data frame that contains the cases and controls

#' @param n_con number of controls to be matched

#' @param cluster_var a variable that contains one case with all available controls to be pooled

#' @param Id_Patient Id of the patient

#' @param total_cont_per_case total number of controls that are available for each case

#' @param case_control a variable containing "case" and "control"

#' @param with_replacement Use replacement or not

#'

#' @import dplyr

#' @return a data frame containing the cases and the corresponding number of controls

#' @export

#'

#' @examples

#' optimal_matching(being_processed, n_con=2, cluster_var=cluster_case,

#' Id_Patient=Patient_Id, total_cont_per_case=total_control_per_case, case_control = case_control)

optimal_matching <- function(total_database, n_con, cluster_var, Id_Patient,

total_cont_per_case, case_control, with_replacement = FALSE) {

if (n_con > max(total_database$total_control_per_case)) {

stop(paste("Number of controls (n_con) should be less than or equal to the total number of controls per case( ",

max(total_database$total_control_per_case), ").", sep=""))

}

if (with_replacement == TRUE) {

cluster_var <- enquo(cluster_var)

Id_Patient <- enquo(Id_Patient)

final_data = total_database %>% group_by((!!cluster_var)) %>% filter(row_number() <= n_con + 1)

return(final_data)

} else {

cluster_var <- enquo(cluster_var)

Id_Patient <- enquo(Id_Patient)

total_cont_per_case <- enquo(total_cont_per_case)

case_control <- enquo(case_control)

dup_con <- 1 # This is needed so as the while loop can work

wave_data <- list() # We create an empty list! Why list? Cause we need to store there all the waves after all iterations!

counter <- 0 # Crucial, important---> see (1), used inside while loop to save data.frames inside wave_data

tmp_database <- total_database # Our original data=total_database should not be overwritten, thus we create a temporary file=tmp_database

waves_round <- list() # Empty list for the rounds one! So, if you want match with 1 control, then 1 round, 3 controls = 3 rounds etc.

while (dup_con > 0) {

# Perform while loop while dup_con > 0.

counter <- counter + 1 # Counter increments by 1, e.g. if we have 8 iterations in round 1 until dup_con=0, then counter=8 and will store all the 8

# 1 iteration # datasets that have been created

datasets <- iterations_per_round(tmp_database, !!cluster_var, !!Id_Patient, !!total_cont_per_case) # We apply iterations_per_round function that returns wave_data, tmp_dataframe and dup_con

wave_data[[counter]] <- datasets[[1]] # Explained in (1)

tmp_database <- datasets[[2]] # Returns the updated tmp_database to perform iterations (when dup_con=0), it is NULL

dup_con <- nrow(datasets[[3]]) # Returns the dup_con until dup_con=0

}

waves_round[[1]] <- do.call("rbind", wave_data) # Explained in (1), crucial because we need 4 we should put it in list. If we need only 1 , then it

# it will return a dataframe of course see (subnote 1)

if (n_con > 1) {

# If we want for example only 1 control, i.e n_con=1, we need to have an else in the bottom, which is final_data<-waves_round[[1]]

waves_round_cont <- list() # Empty list that contains the datasets from each waves_round (only the controls, though that we need to exclude)

for (i_con in 2:n_con) {

# i_con meaning (ith control), starts from 2 (cause 1 was above) until n_con, its value, i.e. 2, 3, 4 is stored in the waves_round[[i_con]] Updated from (subnote 2)

# each time begins from 1 but as i_con increase, this increases too

for (i_wave in 1:length(waves_round)) {

tmp_waves_round <- waves_round[[i_wave]] # Temporary saves waves_round. 1st iteration just waves_round[[1]], 2nd just waves_round[[2]] etc

waves_round_cont[[i_wave]] = tmp_waves_round %>% filter(!!case_control== "control")

}

waves_round_merge <- do.call("rbind", waves_round_cont) # do.call to gather the waves_round_cont

tmp_database <- total_database # Goes back to the original file (cause we need the original one)

tmp_database <- anti_join(tmp_database, waves_round_merge, by = quo_name(Id_Patient))

tmp_database = tmp_database %>% group_by((!!cluster_var)) %>% mutate(!!total_cont_per_case, n() - 1)

dup_con <- 1 # Again the same as in the iterations_per_round but we do it iteratively by using the [[i_con]]

wave_data <- list()

counter <- 0

tmp_database <- tmp_database

while (dup_con > 0) {

counter <- counter + 1

# 1 iteration

datasets <- iterations_per_round(tmp_database, !!cluster_var, !!Id_Patient, !!total_cont_per_case)

wave_data[[counter]] <- datasets[[1]]

tmp_database <- datasets[[2]]

dup_con <- nrow(datasets[[3]])

}

waves_round[[i_con]] <- do.call("rbind", wave_data) # (subnote 2) Starts from 2nd, cause element 1 is already the waves_round[[1]] from above

}

final_data <- waves_round[[1]] # Final step is to keep cases and controls from round 1, but only controls from round 2:4! Cause round 1 by definition has the most cases

for (I_add_con in 2:n_con) {

# So we count from 2:n_con

tmp_waves_round <- waves_round[[I_add_con]] # Temporarary starting from round 2 wave

final_data <- rbind(final_data, tmp_waves_round %>% filter(!!case_control == "control")) # Attach on the waves_round1, only the controls of round 2:4!!

}

} else {

final_data <- waves_round[[1]] # (subnote 1)

}

return(final_data)

}

}
