# Supplementary material for: Fast and optimal algorithm for case-control matching using registry data: application on the antibiotics use of colorectal cancer patients
Source: BMC Med Res Methodol. 2021 Apr 2;21:62. doi: 10.1186/s12874-021-01256-3 (PMC8019172; doi:10.1186/s12874-021-01256-3)
Supplement: Supplementary file 2 — Additional file 2. A detailed vignette of the ccoptimalmatch package using a real-life dataset of INTEGO registry (Flanders, Belgium). The pre-processing phase of the algorithm and its application in the clinical case is provided. [file 12874_2021_1256_MOESM2_ESM.pdf]

Fast and optimal algorithm for case-control matching using registry data: Application on the antibiotics use of colorectal cancer patients.

Pavlos Mamouris; Vahid Nassiri

Matching case-controls in *R* using the `ccoptimalmatch` package

The *R* Environment

R software will be used throughout this vignette. The *R* statistical software is freely available and you may download and install it for Windows, Mac, and Linux systems from: https://www.r-project.org.

R is a user-friendly platform. For example, you can type in the console:

```
1 + 1
#> [1] 2
```

Setup

Load the `ccoptimalmatch` package functions

The `ccoptimalmatch` package functions have been provided to you (Additional file 1). Loading those functions in the R environment, allows the reproducibility of the package.

The main function needed for this tutorial is `optimal_matching`

Datasets

We have two data-sets in this package, namely the "not_processed" and the "being_processed" data-set. Those two data are provided as additional files 3 and 4. The "not_processed" data-set, as the name reveals, is a raw data-set containing the cases, controls, patient_ID and other relevant variables. After different pre-processing steps, we end up to the "being_processed" data, which is the one to use in the algorithm. To see the first 6 rows of the "being_processed" data, enter:

```
#load("yourpath/being_processed.csv")
head(being_processed)
#>   cluster_case     Patient_Id case_control case_ind  JCG entry_year CI age_diff
#> 1       case_1    Patient_101         case        1 2015       2000  7        0
#> 2       case_1 Patient_147179      control        0 2015       2000  2        0
#> 3       case_1 Patient_158654      control        0 2015       2000  6        0
#> 4       case_1 Patient_163420      control        0 2015       2000  4        0
#> 5       case_1 Patient_189606      control        0 2015       2000  4        0
```

```
#> 6       case_1 Patient_200971      control         0 2015       2000 18         0
#>   fup_diff total_control_per_case freq_of_controls
#> 1        0                     25                1
#> 2        0                     25                2
#> 3        0                     25                2
#> 4        0                     25                2
#> 5        0                     25                2
#> 6        0                     25                2
```

If you wish to investigate the data-set and its attributes, then use the help function:

```
help("being_processed")
```

You can directly access a variable within this data frame as follows:

```
being_processed$case_control
```

For example, let us tabulate this variable and investigate the number of cases and controls:

```
table(being_processed$case_control)
#> 
#>    case control 
#>    1637   75473
```

"case_control" is a dummy variable indicating whether the patient is a case or a control. There are 1,637 cases and 75,473 available controls to pool.

Prepare the dataset to be analyzed

Raw data

To see the first 6 rows of the "not_processed" data:

```
#load("yourpath/not_processed.csv")
head(not_processed)
#>   Patient_Id  JCG Birth_Year Practice_Id Gender case_control entry_year foll_up
#> 1  Patient_1 2010       1990  Practice_1      F      control       2008       2
#> 2  Patient_1 2011       1990  Practice_1      F      control       2008       3
#> 3  Patient_2 2012       1942  Practice_2      M      control       2012       0
#> 4  Patient_2 2015       1942  Practice_2      M      control       2012       3
#> 5  Patient_3 2013       1992  Practice_3      M      control       2013       0
#> 6  Patient_3 2014       1992  Practice_3      M      control       2013       1
#>   CI
#> 1  0
#> 2  0
#> 3  0
#> 4  0
#> 5  0
#> 6  0
```

Let us tabulate the "case_control" variable and investigate the number of cases and controls in the "not_processed" data-set this time:

```
table(not_processed$case_control)
#>
#>    case control
#>    1718  654788
```

You can observe that we have 1,718 cases and 654,788 available controls to pool. Controls might be duplicated since a control could appear up to 6 times, from 2010 until 2015. The unique controls to pool are 224,909.

The following steps are necessary to pre-process the "not_processed" data-set in a format that can be used by our algorithm:

Step 1: Exact Matching on several variables

We start by defining the subsets. In order to define the subsets, we filter by the "cases", take the distinct combination of exact variables (Gender, JCG and Practice_Id), and create the new variable "subset". Finally, we select only 4 relevant variables (Gender, JCG, Practice_Id, subset):

```
create_subset <- not_processed %>%
                 filter(case_control =="case") %>%
                 arrange(Practice_Id, Gender, JCG) %>%
                 distinct(Gender, JCG, Practice_Id, .keep_all = TRUE) %>%
                 mutate(subset = 1:n()) %>%
                 select(Gender, JCG, Practice_Id, subset)
```

There were created n (=383) subsets, where a subset is defined as a factorial combination of the exact variables. For example, subset 1 contains females that visited practice 1 in year 2010, subset 2 contains females that visited practice 1 in year 2011, subset 3 contains females that visited practice 1 in year 2012 up to subset n, which is the last factorial combination of the exact variables:

```
head(create_subset)
#>   Gender  JCG Practice_Id subset
#> 1      F 2010  Practice_1      1
#> 2      F 2011  Practice_1      2
#> 3      F 2012  Practice_1      3
#> 4      F 2013  Practice_1      4
#> 5      F 2014  Practice_1      5
#> 6      F 2015  Practice_1      6
```

We merge the data that contains the "subset" variable with the data that contains the cases only:

```
case_with_subset <- not_processed %>%
                         filter(case_control =="case") %>%
                          full_join(create_subset, by = c("Gender", "JCG", "Practice_Id"))
```

We merge the data that contains the "subset" variable with the data that contains the controls only:

```
control_with_subset <- not_processed %>%
                            filter(case_control =="control") %>%
                            right_join(create_subset, by = c("Gender", "JCG", "Practice_Id"))
```

Finally we bind the cases and the controls, which will have now the new variable "subset":

```
not_processed <- rbind(case_with_subset,control_with_subset)
```

Let us tabulate the "case_control" variable again:

```
table(not_processed$case_control)
#> 
#>    case control 
#>    1718  441670
```

As we observe, the number of controls have decreased to 441,670 and the unique controls to 175,018. The gain from exact matching is that by shifting the analysis from one big data-set to several small sub-sets, the computational burden decreases substantially. There were 224,909-175,018 = 49,891 controls that couldn't be matched to any of the cases, thus are excluded.

Step 2: Create artificial observations and select the range of variables

Firstly, we split the data-set in cases and controls and create a variable "cluster_case" to depict the cases separately. The "cluster_case" variable will have as many levels as the total number of cases, i.e. 1,718 in our example. For that purpose, the "cluster_case" will be empty in the controls data-set but have the names of the cases in the cases data-set:

```
bdd_controls <- not_processed[not_processed$case_control=="control",]
bdd_controls$cluster_case <- 0
bdd_cases <- not_processed[not_processed$case_control=="case",]
bdd_cases$cluster_case <- paste("case",1:nrow(bdd_cases),sep = "_")
```

Next, we bind the cases and the controls, which will have now the new variable "cluster_case" and create the variable age:

```
not_processed <- rbind(bdd_cases,bdd_controls)
not_processed$age <- not_processed$JCG-not_processed$Birth_Year
```

After creating the variable "cluster_case", we split again the cases and controls into two different data-sets:

```
bdd_cases <- not_processed[not_processed$case_control=="case",]
bdd_control <- not_processed[not_processed$case_control=="control",]
```

Next, we create an empty data-frame and a unique list of the variable "cluster_case":

```
bdd_temp <- data.frame()
list_p <- unique(bdd_cases$cluster_case)
```

Below it is the loop to generate the pseudo-observations for controls, which will be explained in details. We start by identifying in which subset each case belongs. Next, we check which controls are in the same subset and bind those controls to the case. For example, subset 1 has 2 cases and 2,217 controls. By creating pseudo-observations for controls, subset 1 will have 2 cases and 4,434 controls. Finally, we select the range for the age and follow-up. For demonstration purposes, we decided that an absolute difference of age smaller than 2 is acceptable and that the follow-up time between cases and controls is exact. Since the 2 cases are different in subset 1 in terms of age and follow-up, each case will end up with a different number of controls available to pool:

```r
for(i in 1:length(list_p)){
 temp <- bdd_cases[bdd_cases$cluster_case==list_p[i],]
 subset_identified <- temp$subset
 temp0 <- bdd_control[bdd_control$subset==temp$subset,]
 temp_final <- rbind(temp,temp0)
 temp_final$cluster_case <- list_p[i]
 temp_final=temp_final %>%
           group_by(cluster_case) %>%
           mutate(age_diff = abs(age - age[case_control=="case"]),
           fup_diff = foll_up - foll_up[case_control=="case"])
 temp_final$age_fup <- ifelse(temp_final$age_diff<=2&temp_final$fup_diff==0,"accept","delete")
 temp_final <- temp_final[temp_final$age_fup=="accept",]
 temp_final$age_fup <- NULL
 bdd_temp <- rbind(bdd_temp,temp_final)
}
```

Let us tabulate the "case_control" variable again:

```r
table(bdd_temp$case_control)
#>
#>    case control
#>    1718   75473
```

The number of duplicated controls have decreased to 75,473 and the unique controls to identify are 28,458. Now, all the remaining controls are those that have at most 2 years difference from the case in the same subset, and also have the exact follow up.

Step 3: Create the variables "total controls per case" and "frequency of controls"

We create the variable "total controls per case", which depicts the total pool of controls available for each case. We also create the variable "case_ind" which takes the value 1 if the patient is a case and 0 if the patient is a control. Lastly, we select only relevant variables:

```r
bdd_temp = bdd_temp %>% group_by(cluster_case) %>% mutate(total_control_per_case = n()-1)
bdd_temp$case_ind <- ifelse(bdd_temp$case_control=="case",1,0)
bdd_temp <- subset(bdd_temp, select=c(cluster_case, Patient_Id, case_control, case_ind,
                      JCG, entry_year, CI, age_diff, fup_diff, total_control_per_case))
```

The variable "frequency of controls" depicts how many times a control is assigned to a case:

```r
bdd_temp = bdd_temp %>% group_by(Patient_Id) %>% mutate(freq_of_controls = n())
```

Let us have a glimpse of the data by looking at the 10 first rows:

```r
head(bdd_temp, 10)
#>    cluster_case    Patient_Id case_control case_ind  JCG entry_year CI age_diff
#> 1        case_1   Patient_101         case        1 2015       2000  7        0
#> 2        case_1  Patient_5766      control        0 2015       2000  4        1
#> 3        case_1  Patient_6528      control        0 2015       2000  0        0
#> 4        case_1 Patient_14452      control        0 2015       2000 11        0
#> 5        case_1 Patient_18925      control        0 2015       2000  7        2
```

```
#> 6         case_1 Patient_27762      control         0 2015       2000  1          2
#> 7         case_1 Patient_43794      control         0 2015       2000  6          1
#> 8         case_1 Patient_70288      control         0 2015       2000  2          0
#> 9         case_1 Patient_93520      control         0 2015       2000  6          0
#> 10        case_1 Patient_93923      control         0 2015       2000  5          2
#>    fup_diff total_control_per_case freq_of_controls
#> 1         0                     25                1
#> 2         0                     25                2
#> 3         0                     25                2
#> 4         0                     25                2
#> 5         0                     25                3
#> 6         0                     25                3
#> 7         0                     25                2
#> 8         0                     25                2
#> 9         0                     25                2
#> 10        0                     25                1
```

Some first conclusions can be drawn:

1. For the cases, case_1 has 25 available controls to pool. Suppose that another case has less controls available (e.g. case_2) and the same control is a candidate for both cases with the same characteristcs. This control would be assigned to case_2, which has less controls.
2. For the controls, look at control "patient_5766". His/her frequency is 2, indicating that he/she is available for 2 cases and also that appears in the data-set 2 times. This is important because the controls with the lowest frequency will be matched first, thus leaving the controls with highest frequency available for the next cases.
3. Lastly, we observe that the ordering is not the most optimal yet since the controls are not as close as they should be to the cases in terms of "age-difference" and "frequency of controls", which brings us to the next step.

Step 4: Order variables

Ordering the variables in a correct order is of utter importance. For simplicity, assuming that there are three variables, namely "age-difference", "follow-up difference" and "frequency of controls". The data-set should be ordered by the variables "case", "control", "follow-up difference", "age-difference" and lastly by "frequency of controls". The variable "follow-up difference" appears before "age-difference" since the "follow-up difference" has more weight (importance) than the "age-difference".

```
bdd_temp<-bdd_temp[order(bdd_temp$cluster_case,bdd_temp$case_control,bdd_temp$fup_diff,
                         bdd_temp$age_diff,bdd_temp$freq_of_controls),]
```

By checking the 10 first rows, we can see that the closest controls are ordered after the case, indicating that they are optimal (have the same age-difference). Also, we observe that the "frequency of controls" is ordered which allows the control with the lowest frequency to be matched first:

```
head(bdd_temp, 10)
#>    cluster_case    Patient_Id case_control case_ind  JCG entry_year CI
#> 1        case_1   Patient_101         case        1 2015       2000  7
#> 3        case_1  Patient_6528      control        0 2015       2000  0
#> 4        case_1 Patient_14452      control        0 2015       2000 11
#> 8        case_1 Patient_70288      control        0 2015       2000  2
#> 9        case_1 Patient_93520      control        0 2015       2000  6
```

```
#> 12       case_1 Patient_121560      control         0 2015       2000 15
#> 16       case_1 Patient_147179      control         0 2015       2000  2
#> 18       case_1 Patient_158654      control         0 2015       2000  6
#> 20       case_1 Patient_163420      control         0 2015       2000  4
#> 24       case_1 Patient_189606      control         0 2015       2000  4
#>    age_diff fup_diff total_control_per_case freq_of_controls
#> 1         0        0                     25                1
#> 3         0        0                     25                2
#> 4         0        0                     25                2
#> 8         0        0                     25                2
#> 9         0        0                     25                2
#> 12        0        0                     25                2
#> 16        0        0                     25                2
#> 18        0        0                     25                2
#> 20        0        0                     25                2
#> 24        0        0                     25                2
```

Analysis of the data

We have the data ready to be used for the algorithm. The "optimal_matching" function generates an optimal match between cases and controls in an iterative and computational efficient way. For demonstration purposes, we select 4 controls to match, and we perform the analysis without replacement:

```
final_data <- optimal_matching(bdd_temp, n_con=4, cluster_case, Patient_Id,
                               total_control_per_case, case_control, with_replacement = FALSE)
```

Below we summarise the steps that explain how the algorithm works.

1. Start of round 1. Select one control per case per iteration. We select the first control which is the closest, thus the most optimal.
2. Split between duplicated and unique controls, and assign the duplicated controls to the case that has less available controls to pool.
3. Exclude cases that already have at least 1 control. Also exclude controls that are matched to a case.
4. Repeat steps 1-3 until all cases have at least 1 control (where applicable).
5. End of round 1. Continue to round 2 up to round n, where n is the controls that the user wants to match. If 4 controls are needed, then we have 4 rounds, if 10 controls are needed we have 10 rounds.

We can see the first 20 rows:

```
final_data <- final_data %>% arrange(cluster_case)
head(final_data,20)
#>    cluster_case     Patient_Id case_control case_ind  JCG entry_year CI
#> 1        case_1    Patient_101         case        1 2015       2000  7
#> 2        case_1   Patient_6528      control        0 2015       2000  0
#> 3        case_1  Patient_14452      control        0 2015       2000 11
#> 4        case_1  Patient_70288      control        0 2015       2000  2
#> 5        case_1  Patient_93520      control        0 2015       2000  6
#> 6       case_10    Patient_854         case        1 2012       2000  5
#> 7       case_10    Patient_998      control        0 2012       2000  2
#> 8       case_10 Patient_149215      control        0 2012       2000  3
#> 9       case_10  Patient_11901      control        0 2012       2000  3
```

```
#> 10      case_10  Patient_53402      control        0 2012        2000  0
#> 11     case_100  Patient_12353         case        1 2011        2000  2
#> 12     case_100  Patient_62266      control        0 2011        2000  1
#> 13     case_100  Patient_85104      control        0 2011        2000  1
#> 14     case_100 Patient_106331      control        0 2011        2000  2
#> 15     case_100 Patient_153503      control        0 2011        2000  1
#> 16    case_1000 Patient_129455         case        1 2013        2000  5
#> 17    case_1000 Patient_128709      control        0 2013        2000  8
#> 18    case_1000   Patient_5004      control        0 2013        2000  3
#> 19    case_1000  Patient_17452      control        0 2013        2000  2
#> 20    case_1000  Patient_33405      control        0 2013        2000  3
#>    age_diff fup_diff total_control_per_case freq_of_controls n() - 1
#> 1         0        0                     25                1      NA
#> 2         0        0                     25                2      NA
#> 3         0        0                     25                2      23
#> 4         0        0                     25                2      21
#> 5         0        0                     25                2      19
#> 6         0        0                     31                1      NA
#> 7         0        0                     31                3      NA
#> 8         0        0                     31                3      28
#> 9         1        0                     31                3      25
#> 10        1        0                     31                3      22
#> 11        0        0                     63                1      NA
#> 12        0        0                     63                4      NA
#> 13        0        0                     63                4      60
#> 14        0        0                     63                4      56
#> 15        0        0                     63                4      52
#> 16        0        0                     27                1      NA
#> 17        0        0                     27                1      NA
#> 18        1        0                     27                1      26
#> 19        1        0                     27                1      25
#> 20        1        0                     27                1      24
```

If we want to see how many controls are matched for each case, then:

```
final_data = final_data %>% group_by(cluster_case) %>% mutate(total_control_matched = n()-1)
table(final_data$case_control,final_data$total_control_matched)
#>
#>              1    2    3    4
#>   case      82   79   70 1405
#>   control   82  158  210 5620
```

82 cases have only 1 control, 79 cases have 2 controls, 70 cases have 3 controls and finally 1405 cases have 4 controls, using the criteria above.

Extensions and Summary

In the clinical case described in the previous sections, we used 3 exact variables (Gender, JCG, Practice_Id), age and follow-up. This algorithm is very flexible in accommodating even more continuous and exact variables, and as a matter of fact, the more criteria are added, the less the computational burden is. It is very useful to operate different scenarios of matching by adjusting the age range, the follow-up time and the Comorbidity Index.

The user (epidemiologist, researcher) has available a 1:n case-control matching algorithm in an optimal, efficient, and fast way using a multi-step (iterative) procedure for traditional and nested case-control studies.
