# Supplementary material for: Fast and optimal algorithm for case-control matching using registry data: application on the antibiotics use of colorectal cancer patients
Source: BMC Med Res Methodol. 2021 Apr 2;21:62. doi: 10.1186/s12874-021-01256-3 (PMC8019172; doi:10.1186/s12874-021-01256-3)
Supplement: Supplementary file 5 — Additional file 5. ORs and CIs for a factorial combination of variables using different control sampling. 72 Scenarios displaying the ORs and the corresponding CIs for a factorial combination of variables using with and without replacement of controls. [file 12874_2021_1256_MOESM5_ESM.docx]

**Fast and optimal algorithm for case-control matching using registry data: Application on the antibiotics use of colorectal cancer patients**

Pavlos Mamouris^1*^; Vahid Nassiri^2^ ; Geert Molenberghs^3,4;^ Marjan van den Akker^1,5,6;^ Joep van den Meer^1^; Bert Vaes^1^

**ORs and CIs for a factorial combination of variables using different control sampling**

|  |  |  |  |  |  |  | cases having 1-4 controls | | | |  |  |  |
| --- | --- | --- | --- | --- | --- | --- | --- | --- | --- | --- | --- | --- | --- |
| Scenarios | Replacement | Follow up | Age | CI | cases | controls | con1 | con2 | con3 | con4 | OR | 95%  LCI | 95%  UCI |
| 1 | Without | exact | exact | - | 1415 | 4632 | 205 | 141 | 131 | 938 | 1.25 | 1.07 | 1.46 |
| 2 | Without | exact | [-1,1] | - | 1590 | 5694 | 118 | 108 | 96 | 1268 | 1.34 | 1.16 | 1.54 |
| 3 | Without | exact | [-2,2] | - | 1636 | 6070 | 82 | 79 | 70 | 1405 | 1.38 | 1.20 | 1.59 |
| 4 | Without | exact | [-3,3] | - | 1666 | 6280 | 61 | 68 | 65 | 1472 | 1.40 | 1.22 | 1.60 |
| 5 | Without | exact | [-4,4] | - | 1683 | 6416 | 48 | 58 | 56 | 1521 | 1.42 | 1.24 | 1.63 |
| 6 | Without | exact | [-5,5] | - | 1692 | 6506 | 41 | 46 | 47 | 1558 | 1.43 | 1.25 | 1.63 |
| 7 | Without | trimmed | exact | - | 1689 | 6132 | 91 | 107 | 137 | 1354 | 1.28 | 1.12 | 1.47 |
| 8 | Without | trimmed | [-1,1] | - | 1711 | 6732 | 6 | 19 | 56 | 1630 | 1.39 | 1.21 | 1.58 |
| 9 | Without | trimmed | [-2,2] | - | 1713 | 6815 | 2 | 7 | 17 | 1687 | 1.45 | 1.27 | 1.66 |
| 10 | Without | trimmed | [-3,3] | - | 1715 | 6835 | 1 | 4 | 14 | 1696 | 1.46 | 1.28 | 1.67 |
| 11 | Without | trimmed | [-4,4] | - | 1716 | 6849 | 1 | 2 | 8 | 1705 | 1.49 | 1.30 | 1.70 |
| 12 | Without | trimmed | [-5,5] | - | 1716 | 6855 | 0 | 2 | 5 | 1709 | 1.48 | 1.29 | 1.69 |
| 13 | Without | exact | exact | [-1,1] | 1239 | 3569 | 275 | 203 | 156 | 605 | 1.06 | 0.89 | 1.26 |
| 14 | Without | exact | [-1,1] | [-1,1] | 1481 | 4986 | 172 | 168 | 86 | 1055 | 1.16 | 0.99 | 1.35 |
| 15 | Without | exact | [-2,2] | [-1,1] | 1550 | 5477 | 123 | 122 | 110 | 1195 | 1.17 | 1.01 | 1.35 |
| 16 | Without | exact | [-3,3] | [-1,1] | 1590 | 5748 | 112 | 91 | 94 | 1293 | 1.16 | 1.00 | 1.34 |
| 17 | Without | exact | [-4,4] | [-1,1] | 1615 | 5940 | 98 | 78 | 70 | 1369 | 1.16 | 1.00 | 1.34 |
| 18 | Without | exact | [-5,5] | [-1,1] | 1628 | 6070 | 78 | 70 | 68 | 1412 | 1.15 | 1.00 | 1.33 |
| 19 | Without | trimmed | exact | [-1,1] | 1552 | 4932 | 221 | 210 | 193 | 928 | 1.15 | 0.99 | 1.34 |
| 20 | Without | trimmed | [-1,1] | [-1,1] | 1678 | 6241 | 62 | 101 | 83 | 1432 | 1.19 | 1.03 | 1.37 |
| 21 | Without | trimmed | [-2,2] | [-1,1] | 1691 | 6538 | 21 | 46 | 71 | 1553 | 1.22 | 1.06 | 1.40 |
| 22 | Without | trimmed | [-3,3] | [-1,1] | 1700 | 6648 | 16 | 30 | 44 | 1610 | 1.26 | 1.09 | 1.45 |
| 23 | Without | trimmed | [-4,4] | [-1,1] | 1703 | 6720 | 10 | 15 | 32 | 1646 | 1.30 | 1.13 | 1.49 |
| 24 | Without | trimmed | [-5,5] | [-1,1] | 1705 | 6749 | 10 | 10 | 21 | 1664 | 1.26 | 1.10 | 1.45 |
| 25 | Without | exact | exact | exact cat | 1241 | 3615 | 275 | 186 | 152 | 628 | 1.10 | 0.93 | 1.31 |
| 26 | Without | exact | [-1,1] | exact cat | 1481 | 4958 | 189 | 152 | 95 | 1045 | 1.14 | 0.98 | 1.33 |
| 27 | Without | exact | [-2,2] | exact cat | 1546 | 5443 | 135 | 117 | 102 | 1192 | 1.16 | 1.00 | 1.34 |
| 28 | Without | exact | [-3,3] | exact cat | 1591 | 5718 | 121 | 95 | 93 | 1282 | 1.15 | 1.00 | 1.33 |
| 29 | Without | exact | [-4,4] | exact cat | 1617 | 5904 | 106 | 76 | 94 | 1341 | 1.17 | 1.01 | 1.35 |
| 30 | Without | exact | [-5,5] | exact cat | 1631 | 6036 | 91 | 69 | 77 | 1394 | 1.17 | 1.01 | 1.35 |
| 31 | Without | trimmed | exact | exact cat | 1554 | 4972 | 215 | 195 | 209 | 935 | 1.19 | 1.02 | 1.38 |
| 32 | Without | trimmed | [-1,1] | exact cat | 1689 | 6238 | 67 | 111 | 95 | 1416 | 1.20 | 1.04 | 1.38 |
| 33 | Without | trimmed | [-2,2] | exact cat | 1704 | 6556 | 31 | 46 | 75 | 1552 | 1.19 | 1.04 | 1.37 |
| 34 | Without | trimmed | [-3,3] | exact cat | 1711 | 6667 | 20 | 32 | 53 | 1606 | 1.21 | 1.05 | 1.39 |
| 35 | Without | trimmed | [-4,4] | exact cat | 1713 | 6729 | 16 | 16 | 43 | 1638 | 1.21 | 1.06 | 1.39 |
| 36 | Without | trimmed | [-5,5] | exact cat | 1713 | 6761 | 14 | 9 | 31 | 1659 | 1.20 | 1.04 | 1.37 |
| 1 | With | exact | exact | - | 1419 | 4774 | 186 | 127 | 90 | 1016 | 1.22 | 1.04 | 1.42 |
| 2 | With | exact | [-1,1] | - | 1590 | 5763 | 109 | 96 | 78 | 1307 | 1.30 | 1.13 | 1.50 |
| 3 | With | exact | [-2,2] | - | 1637 | 6117 | 80 | 68 | 55 | 1434 | 1.38 | 1.20 | 1.58 |
| 4 | With | exact | [-3,3] | - | 1666 | 6315 | 55 | 70 | 44 | 1497 | 1.38 | 1.21 | 1.59 |
| 5 | With | exact | [-4,4] | - | 1683 | 6442 | 42 | 57 | 50 | 1534 | 1.40 | 1.22 | 1.61 |
| 6 | With | exact | [-5,5] | - | 1693 | 6527 | 41 | 39 | 44 | 1569 | 1.42 | 1.24 | 1.63 |
| 7 | With | trimmed | exact | - | 1693 | 6363 | 60 | 74 | 81 | 1478 | 1.21 | 1.05 | 1.39 |
| 8 | With | trimmed | [-1,1] | - | 1711 | 6809 | 4 | 6 | 11 | 1690 | 1.35 | 1.19 | 1.55 |
| 9 | With | trimmed | [-2,2] | - | 1713 | 6840 | 2 | 2 | 2 | 1707 | 1.44 | 1.26 | 1.65 |
| 10 | With | trimmed | [-3,3] | - | 1715 | 6850 | 1 | 3 | 1 | 1710 | 1.45 | 1.27 | 1.66 |
| 11 | With | trimmed | [-4,4] | - | 1716 | 6855 | 1 | 2 | 2 | 1711 | 1.47 | 1.28 | 1.68 |
| 12 | With | trimmed | [-5,5] | - | 1716 | 6857 | 0 | 2 | 5 | 1709 | 1.48 | 1.29 | 1.69 |
| 13 | With | exact | exact | [-1,1] | 1245 | 3715 | 263 | 174 | 128 | 680 | 1.04 | 0.88 | 1.23 |
| 14 | With | exact | [-1,1] | [-1,1] | 1482 | 5084 | 163 | 142 | 71 | 1106 | 1.17 | 1.01 | 1.37 |
| 15 | With | exact | [-2,2] | [-1,1] | 1550 | 5544 | 118 | 109 | 84 | 1239 | 1.18 | 1.01 | 1.36 |
| 16 | With | exact | [-3,3] | [-1,1] | 1590 | 5805 | 107 | 81 | 72 | 1330 | 1.18 | 1.02 | 1.37 |
| 17 | With | exact | [-4,4] | [-1,1] | 1615 | 5980 | 92 | 77 | 50 | 1396 | 1.20 | 1.04 | 1.39 |
| 18 | With | exact | [-5,5] | [-1,1] | 1628 | 6100 | 75 | 64 | 59 | 1430 | 1.16 | 1.00 | 1.34 |
| 19 | With | trimmed | exact | [-1,1] | 1562 | 5153 | 194 | 174 | 165 | 1029 | 1.12 | 0.97 | 1.30 |
| 20 | With | trimmed | [-1,1] | [-1,1] | 1679 | 6369 | 51 | 68 | 58 | 1502 | 1.17 | 1.02 | 1.34 |
| 21 | With | trimmed | [-2,2] | [-1,1] | 1691 | 6605 | 19 | 31 | 40 | 1601 | 1.21 | 1.05 | 1.39 |
| 22 | With | trimmed | [-3,3] | [-1,1] | 1700 | 6697 | 14 | 19 | 23 | 1644 | 1.27 | 1.10 | 1.46 |
| 23 | With | trimmed | [-4,4] | [-1,1] | 1703 | 6748 | 9 | 12 | 13 | 1669 | 1.32 | 1.15 | 1.51 |
| 24 | With | trimmed | [-5,5] | [-1,1] | 1705 | 6771 | 9 | 6 | 10 | 1680 | 1.26 | 1.10 | 1.45 |
| 25 | With | exact | exact | exact cat | 1248 | 3786 | 256 | 154 | 130 | 708 | 1.08 | 0.91 | 1.29 |
| 26 | With | exact | [-1,1] | exact cat | 1481 | 5074 | 173 | 120 | 91 | 1097 | 1.12 | 0.97 | 1.31 |
| 27 | With | exact | [-2,2] | exact cat | 1546 | 5522 | 129 | 98 | 79 | 1240 | 1.15 | 0.99 | 1.33 |
| 28 | With | exact | [-3,3] | exact cat | 1592 | 5786 | 120 | 83 | 56 | 1333 | 1.14 | 0.99 | 1.32 |
| 29 | With | exact | [-4,4] | exact cat | 1618 | 5957 | 105 | 68 | 64 | 1381 | 1.17 | 1.01 | 1.35 |
| 30 | With | exact | [-5,5] | exact cat | 1632 | 6075 | 89 | 66 | 54 | 1423 | 1.16 | 1.01 | 1.34 |
| 31 | With | trimmed | exact | exact cat | 1562 | 5220 | 179 | 159 | 173 | 1051 | 1.14 | 0.98 | 1.32 |
| 32 | With | trimmed | [-1,1] | exact cat | 1690 | 6406 | 53 | 59 | 77 | 1501 | 1.14 | 0.99 | 1.31 |
| 33 | With | trimmed | [-2,2] | exact cat | 1704 | 6646 | 29 | 24 | 35 | 1616 | 1.16 | 1.01 | 1.33 |
| 34 | With | trimmed | [-3,3] | exact cat | 1711 | 6734 | 20 | 16 | 18 | 1657 | 1.20 | 1.04 | 1.37 |
| 35 | With | trimmed | [-4,4] | exact cat | 1713 | 6775 | 16 | 7 | 15 | 1675 | 1.20 | 1.05 | 1.38 |
| 36 | With | trimmed | [-5,5] | exact cat | 1713 | 6794 | 13 | 5 | 9 | 1686 | 1.17 | 1.02 | 1.35 |
